# Supplementary material for: Impact of Covid-19 pandemic on obstetric fistula repair program in Zimbabwe
Source: PLoS One. 2021 Apr 1;16(4):e0249398. doi: 10.1371/journal.pone.0249398 (PMC8016295; doi:10.1371/journal.pone.0249398)
Supplement: S1 File — (DOCX) [file pone.0249398.s001.docx]

# **Appendix 2: Interview guide for key informants**

**Impact of Covid-19 pandemic on obstetric fistula repair program in Zimbabwe**

My name is Chipo Chimamise a DPhil Public Health student with the Africa University. I am conducting a research project on the **Impact of Covid-19 pandemic on obstetric fistula repair program in Zimbabwe.**

Before we start, I would like to remind you that there are no right or wrong answers in this discussion. I am interested in knowing your experiences in relation to obstetric fistula programming at your institutions, so please feel free to answer truthfully and to share your experiences and opinions. If I ask something you are not comfortable to answer, please tell me and we will skip it.

|  | Variable | Answers |
| --- | --- | --- |
|  | Name of hospital |  |
|  | Title/post of interviewee |  |
|  | Province |  |
|  | Date of interview |  |
|  | Year of starting fistula repair program |  |

1. Let us talk about obstetric fistula programming here at your institution. Tell me when this hospital started the repair program at a public health scale. Before then, did the hospital conduct fistula repairs? If so, tell me the average number of fistula cases which used to be repaired here per year.
2. Let us look at the fistula repair program to compare its performance here at your hospital comparing the years 2019 and 2020. Probes:

- *Tell me about the numbers of women repaired*
- *The easiness of running the fistula camps*

1. Now let’s discus about challenges faced by the program here at your hospital comparing 2019 and 2020

Probes:

- *Have you had any Covid-19 patients?*
- *How prepared were you for the pandemic/*
- *Has the pandemic caused your hospital to make major restructuring, reassignement of staff and spaces or supplies?*

1. Regarding the obstetric fistula repair program at this hospital, what were the changes, constraints or advantages/disadvantages which were brought about by the Covid-19 pandemic.

Probes: *Talk about*

- *Human resources reassignments*
- *Reassignment of hospital wards and other work spaces*
- *Equipment, commodities and supplies re allocation*
- *Continuation of other services which are not Covid-19 related, emergencies and non-emergencies*

1. Tell me your opinion on the state of preparedness your hospital was for Covid-19 pandemic and what you think should be done going forward
2. Any other comments you have on fistula programming in the context of Covid-19 pandemic and other emergency situations

Thank you for your participation
