# Supplementary material for: Impact of Covid-19 pandemic on obstetric fistula repair program in Zimbabwe
Source: PLoS One. 2021 Apr 1;16(4):e0249398. doi: 10.1371/journal.pone.0249398 (PMC8016295; doi:10.1371/journal.pone.0249398)
Supplement: Attachment — Submitted filename: PONE-D-21-05832_reviewer(1).pdf [file pone.0249398.s003.pdf]

PLOS ONE

Impact of Covid-19 pandemic on obstetric fistula repair programming and characteristics of Obstetric fistula repair camp attendees at Mashoko Christian Hospital in Zimbabwe

--Manuscript Draft--

**Manuscript Number:**	PONE-D-21-05832
**Article Type:**	Research Article
**Full Title:**	Impact of Covid-19 pandemic on obstetric fistula repair programming and characteristics of Obstetric fistula repair camp attendees at Mashoko Christian Hospital in Zimbabwe
**Short Title:**	Effecst of Covid-19 pandemic on obstetric fistula programming in Zimbabwe
**Corresponding Author:**	CHIPO CHIMAMISE<br>Ministry of Health and Child Care Zimbabwe<br>Harare, Harare ZIMBABWE
**Keywords:**	Obstetric fistula programming, Covid-19 pandemic effects, characteristics, fistula survivors, Zimbabwe
**Abstract:**	The advent of Covid-19 pandemic adversely affected many programs worldwide, public health, including programming for obstetric fistula were not spared. Obstetric fistula is an abnormal connection between the vagina and the bladder or the rectum resulting from obstetric causes, mainly prolonged obstructed labour. Zimbabwe has two obstetric fistula repair centers. Because the program uses specialist surgeons from outside the country, the repairs are organized in quarterly camps with a target to repair 90 women per quarter. This study aimed at assessing the impact of restrictions on movement and gathering of people brought about by the Cocid-19 pandemic and to characterize participants of the camp which was held in the midst of the Covid-19 pandemic at Mashoko Hospital. Specifically it looked at how Covid-19 pandemic affected programming for obstetric fistula repair and characterized participants of the fistula camp held in November to December 2020 at one of the repair centers. A review of the dataset and surgical log sheets for the camp and national obstetric fistula dataset was conducted. Variables of interest were extracted onto an excel spreadsheet and analyzed for frequencies and proportions. Data were presented in charts, tables and narratives. The study noted that Covid-19 pandemic negatively affected performance of fistula repairs greatly with only 25 women repaired in 2020 as compared to 313 in 2019. Ninety women were called to come for repairs but 52 did not manage to attend due to reasons related to the restriction of the Covid-19 pandemic lockdown. Two thirds of those women suffered from urinary incontinence while the other third had fecal incontinence. The successful repair rate was 92%. This study concluded that the pandemic greatly affected programming of fistula repair in the country and recommended the Ministry of Health and Child Care to institute measures to resume programming as soon as the situation allows.
**Order of Authors:**	CHIPO CHIMAMISE
	Stephen Peter Munjanja
	Mazvita Machinga
	Iris Shiripinda
**Additional Information:**	
**Question**	**Response**
**Financial Disclosure**<br><br>Enter a financial disclosure statement that describes the sources of funding for the work included in this submission. Review the submission guidelines for detailed	The authors received no specific funding for this work

Powered by Editorial Manager® and ProduXion Manager® from Aries Systems Corporation

requirements. View published research articles from *PLOS ONE* for specific examples.<br><br>This statement is required for submission and **will appear in the published article** if the submission is accepted. Please make sure it is accurate.<br><br>**Unfunded studies**<br>Enter: *The author(s) received no specific funding for this work.*<br><br>**Funded studies**<br>Enter a statement with the following details:<br>• Initials of the authors who received each award<br>• Grant numbers awarded to each author<br>• The full name of each funder<br>• URL of each funder website<br>• Did the sponsors or funders play any role in the study design, data collection and analysis, decision to publish, or preparation of the manuscript?<br>• **NO** - Include this sentence at the end of your statement: *The funders had no role in study design, data collection and analysis, decision to publish, or preparation of the manuscript.*<br>• **YES** - Specify the role(s) played.<br><br>* typeset	
**Competing Interests**<br><br>Use the instructions below to enter a competing interest statement for this submission. On behalf of all authors, disclose any competing interests that could be perceived to bias this work—acknowledging all financial support and any other relevant financial or non-financial competing interests.<br><br>This statement is required for submission and **will appear in the published article** if the submission is accepted. Please make sure it is accurate and that any funding sources listed in your Funding Information later in the submission form are also declared in your Financial Disclosure	The authors declare that no competing interests exist

statement.<br><br>View published research articles from *PLOS ONE* for specific examples.<br><br>**NO authors have competing interests**<br><br>Enter: *The authors have declared that no competing interests exist.*<br><br>**Authors with competing interests**<br><br>Enter competing interest details beginning with this statement:<br><br>*I have read the journal's policy and the authors of this manuscript have the following competing interests: [insert competing interests here]*<br><br>* typeset	
**Ethics Statement**<br><br>Enter an ethics statement for this submission. This statement is required if the study involved:<br><br>• Human participants<br>• Human specimens or tissue<br>• Vertebrate animals or cephalopods<br>• Vertebrate embryos or tissues<br>• Field research<br><br>Write "N/A" if the submission does not require an ethics statement.<br><br>General guidance is provided below. Consult the submission guidelines for detailed instructions. **Make sure that all information entered here is included in the Methods section of the manuscript.**	This article is part of a lager study documenting obstetric fistula experiences in Zimbabwe and was approved by the Medical Research Council of Zimbabwe under license: MRCZA2525. Key informants gave written informed consent. All participants gave written informed consent. Data for the fistula camp attendees were analyzed anonymously, no personal identifying information was used.

Powered by Editorial Manager® and ProduXion Manager® from Aries Systems Corporation

**Format for specific study types**<br><br>**Human Subject Research (involving human participants and/or tissue)**<br>• Give the name of the institutional review board or ethics committee that approved the study<br>• Include the approval number and/or a statement indicating approval of this research<br>• Indicate the form of consent obtained (written/oral) or the reason that consent was not obtained (e.g. the data were analyzed anonymously)<br><br>**Animal Research (involving vertebrate animals, embryos or tissues)**<br>• Provide the name of the Institutional Animal Care and Use Committee (IACUC) or other relevant ethics board that reviewed the study protocol, and indicate whether they approved this research or granted a formal waiver of ethical approval<br>• Include an approval number if one was obtained<br>• If the study involved *non-human primates*, add *additional details* about animal welfare and steps taken to ameliorate suffering<br>• If anesthesia, euthanasia, or any kind of animal sacrifice is part of the study, include briefly which substances and/or methods were applied<br><br>**Field Research**<br><br>Include the following details if this study involves the collection of plant, animal, or other materials from a natural setting:<br>• Field permit number<br>• Name of the institution or relevant body that granted permission	
**Data Availability**<br><br>Authors are required to make all data underlying the findings described fully available, without restriction, and from the time of publication. PLOS allows rare exceptions to address legal and ethical concerns. See the PLOS Data Policy and FAQ for detailed information.	Yes - all data are fully available without restriction

Powered by Editorial Manager® and ProduXion Manager® from Aries Systems Corporation

A Data Availability Statement describing where the data can be found is required at submission. Your answers to this question constitute the Data Availability Statement and **will be published in the article**, if accepted.<br><br>**Important:** Stating 'data available on request from the author' is not sufficient. If your data are only available upon request, select 'No' for the first question and explain your exceptional situation in the text box.<br><br>Do the authors confirm that all data underlying the findings described in their manuscript are fully available without restriction?	
**Describe where the data may be found in full sentences. If you are copying our sample text, replace any instances of XXX with the appropriate details.**<br><br>• If the data are **held or will be held in a public repository**, include URLs, accession numbers or DOIs. If this information will only be available after acceptance, indicate this by ticking the box below. For example: *All XXX files are available from the XXX database (accession number(s) XXX, XXX.).*<br>• If the data are all contained **within the manuscript and/or Supporting Information files**, enter the following: *All relevant data are within the manuscript and its Supporting Information files.*<br>• If neither of these applies but you are able to provide **details of access elsewhere**, with or without limitations, please do so. For example:<br><br>*Data cannot be shared publicly because of [XXX]. Data are available from the XXX Institutional Data Access / Ethics Committee (contact via XXX) for researchers who meet the criteria for access to confidential data.*<br><br>*The data underlying the results presented in the study are available from (include the name of the third party*	All relevant data are within the manuscript and its Supporting Information files.

and contact information or URL).<br>• This text is appropriate if the data are owned by a third party and authors do not have permission to share the data.<br><br>* typeset	
Additional data availability information:	

Impact of Covid-19 pandemic on obstetric fistula repair programming and characteristics of Obstetric fistula repair camp attendees at Mashoko Christian Hospital in Zimbabwe

Short title: Effects of Covid-19 pandemic on obstetric fistula programming in Zimbabwe

Chipo Chimamise[1], Stephen P. Munjanja[2¶], Mazvita Machinga[1¶] and Iris Shiripinda[1¶]

[1](Department of Health Sciences, College of Health, Agriculture and Natural Sciences, Africa University, Mutare, Zimbabwe)

[2](Department of Obstetrics and Gyynaecology, College of Health Sciences, University of Zimbabwe, Harare, Zimbabwe)

*Corresponding author

Email: chipochimamise@gmail.com (CC)

¶These authors contributed equally to this work.

Abstract

The advent of Covid-19 pandemic adversely affected many programs worldwide, public health, including programming for obstetric fistula were not spared. Obstetric fistula is an abnormal connection between the vagina and the bladder or the rectum resulting from obstetric causes, mainly prolonged obstructed labour. Zimbabwe has two obstetric fistula repair centers. Because the program uses specialist surgeons from outside the country, the repairs are organized in quarterly camps with a target to repair 90 women per quarter. This study aimed at assessing the impact of restrictions on movement and gathering of people brought about by the Cocid-19 pandemic and to characterize participants of the camp which was held in the midst of the Covid-19 pandemic at Mashoko Hospital. Specifically it looked at how Covid-19 pandemic affected programming for obstetric fistula repair and characterized participants of the fistula camp held in November to December 2020 at one of the repair centers. A review of the dataset and surgical log sheets for the camp and national obstetric fistula dataset was conducted. Variables of interest were extracted onto an excel spreadsheet and analyzed for frequencies and proportions. Data were presented in charts, tables and narratives. The study noted that Covid-19 pandemic negatively affected performance of fistula repairs greatly with only 25 women repaired in 2020 as compared to 313 in 2019. Ninety women were called to come for repairs but 52 did not manage to attend due to reasons related to the restriction of the Covid-19 pandemic lockdown. Two thirds of those women suffered from urinary incontinence while the other third had fecal incontinence. The successful repair rate was 92%. This study concluded that the pandemic greatly affected programming of fistula repair in the country and recommended the Ministry of Health and Child Care to institute measures to resume programming as soon as the situation allows.

Key words: Obstetric fistula programming, Covid-19 pandemic, characteristics, fistula survivors

Introduction

Obstetric fistula, an abnormal connection between the vagina and either the bladder (vesicovaginal fistula) or the rectum (rectovaginal fistula) presents untold suffering to affected women [1]. The condition results mostly from ischemic trauma caused by prolonged and obstructed labour [2]. It affects all aspects of the woman's life including physical life, spiritual, emotional, social and economic life [3]. Worldwide, obstetric fistula affects between 500,00 and 100,000 women every year, with Africa and Asia bearing the highest burden [4].

In Zimbabwe, the actual burden of obstetric fistula is not known. The country started an obstetric fistula repair program as a public health intervention in August 2015 at one provincial hospital. Before then, obstetric fistula repair was mainly available at private hospital and it was out of reach of many financially compromised women. As efforts to strengthen the fistula program, the Ministry of Health and Child care piloted a community fistula surveillance system in Manicaland province which is being driven by community based workers.

The program has since been expanded to one more hospital, Mashoko Christian Hospital in Masvingo province and since then more than 700 obstetric fistula survivors have been repaired. Mashoko hospital has up to now conducted five camps. Since the program mostly makes use of visiting surgeons from other countries, fistula repair activities are organized periodically, patients are identified and put on a waiting list.

Fistula repair dates are scheduled usually on a quarterly basis and patients are called for repair on the scheduled dates, this is termed a fistula repair camp.

Zimbabwe registered its first Covid-19 case on the 23rd of March 2020 and the country went into a lockdown with stringent regulations which restricted movement and gatherings. This affected the fistula camp which was organized to start that week where a total of 90 women were expected to be repaired at the two fistula repair sites. The lockdown regulations were relaxed in July to allow limited gatherings and more free movement of people. But Chinhoyi Provincial Hospital was not open for fistula camps mainly because staff and resources had been drained due to the Covid-19 pandemic and part of the hospital was designated as a Covid-19 wing, so there was no space to hold a fistula camp. Mashoko hospital called for a camp which was scheduled for November to December 2020 even though there was limited freedom on movement of people and other infection prevention and control measures.

To strengthen the fistula program, it is crucial to have information on beneficiaries of the program so that interventions are tailored to the needs of the beneficiaries, such as their ages, when they develop the fistula and whether the program is effective in bringing relief from their suffering.

The aim of this study was to document effects of the Covid-19 pandemic on the fistula repair program in Zimbabwe and to characterize participants of the obstetric fistula repair camp held in November to December 2020 at Mashoko Christian Hospital. Its objectives were to determine

the extent to which Covid-19 pandemic affected fistula repair programing, to determine demographic characteristics of the camp's participants in relation to where they came from, who referred them for the camp, their problems and surgical outcomes.

Methods

Study design and study setting

A retrospective cross sectional study was conducted using analysis of program data on the obstetric fistula repair camp conducted at Mashoko Christian Hospital and national obstetric fistula database.

Study datasets

The dataset contains information and data elements on name of patient, village, district and province of origin, age, period staying with fistula, who referred the patient, whether it is a new or old patient, number of previous fistula repairs, type of injury and type of fistula, surgical diagnosis and surgical procedure performed, surgical outcome and number of days in hospital, among others. These were the data elements needed for this study. Personal identifying data elements such as name, hospital number, contact details and national identity number, were removed from the dataset before analysis to maintain confidentiality. The national fistula program dataset was also analyzed to assess the trends in fistula repairs per quarter in the year compared to the same period the previous year.

Sampling and sample size

Records of all the thirty patients who attended the November-December 2020 fistula repair camp at Mashoko Hospital were purposively included in the study. The national fistula repair dataset was also analyzed for camp attendance for 2019 and 2020.

Data collection

An excel spreadsheet with data elements of interest was prepared and data were extracted from the surgical log sheet onto the spreadsheet. Completeness of the data was assured during the process of data extraction by cross checking the log sheet against the patient clinical records. Key informant interviews were also held with the hospital superintendents of the fistula repair hospitals and the National Obstetric Fistula Officer to get information on program implementation in the context of Covid-19.

Data analysis and presentation

Since the volume of the data was relatively small, data were analyzed using the excel spreadsheet for frequencies and proportions. Information from the key informant interviews were analyzed manually for content. The data were presented in tables, figures and narratives.

Ethical considerations

This article is part of a lager study approved by the Medical Research Council of Zimbabwe under license MRCZ A2525. This study mainly used a records review approach, reviewing the history of how the patients were identified and other demographic data from their medical records excluding their personal identifications. Written informed consent was obtained from all

study participants. Key informants gave written informed consent. Confidentiality was maintained throughout the study.

Results

The Covid-19 pandemic hindered the holding of fistula repair camps in 2020. There was only one camp held with only 25 fistula cases and 5 perineal tears repaired. Fig 1 below illustrates the comparison of fistula cases repaired in 2020 when Covid-19 resulted in restrictions on movement and gathering of people against 2019 when such restrictions were not there.

Fig 1: Trends in fistula camp attendance before and during the Covid-19 pandemic

A total of 90 women with urine and/or fecal incontinence were called for the repair camp, 82 confirmed that they were coming. Of these 52 did not reach the facility because of various reasons related to Covid-19 lockdown restrictions. Fig 2 below shows the numbers of women called for the camp, those who could not come for various reasons and those who actually came.

Fig 2: Cascade of the process from being called to attend the camp to actual attendance

Reasons given for not reporting for the camp included:

- Could not get travel authorization documents - 16
- Afraid of catching Covid-19 – 8
- Afraid of law enforcement agents - 15
- Were turned back by law enforcement agents at check-points - 13

This left only 30 women who actually attended the camp. This is against the initial plan of repairing 90 women. Fig 3 below shows the distribution of fistula camp attendees by province.

Fig 3: Distribution of participants by province of origin

Fig 3 shows that half (15 of the 30) of study participants came from Manicaland province. On arrival, all women were screened for Covid-19 infection symptomatically. They had temperature checks and asked questions on whether they had symptoms. They had a rapid test for the infection. Three of the thirty women got a positive rapid test result. They were immediately isolated and specimens for DNA PCR tests were collected and sent for testing. These came out negative and so the women were removed from isolation.

Measures for infection prevention and control were strictly observed, i.e. frequent handwashing and sanitization, social distancing and wearing of facemasks. The women were admitted in batches of five per day and a ward which used to accommodate 30 patients was able to accommodate only 10.

Other demographic data are presented in Table 1 below.

Table 1: Demographic characteristics of participants

	**Age groups**	
15-19	20-24	25+
6	7	17
	**Time staying with incontinence**	
<1year	1-5 years	>5 years
11	8	11
	**Type of referral**	
Health facility	Community-based workers	Radio
4	22	4

Table 1 shows that adolescents and young women below the age of 25 contributed a significant proportion (43%) of affected women with 6 participants being below 20 years. The oldest was 73 years old and the youngest was 17. The majority (19 women) of the participants developed the incontinence problem in the 5 years preceding this study. The most recent fistula had a duration of 3 months while the longest duration was 39 years. Most (22 women) of the study participants were referred by community based workers.

Of the 30 women who attended the fistula repair camp, five had fecal incontinence due to 3rd and 4th degree perineal tears and 25 had fistula.

Table 2: Type of incontinence and type of injury

Type of incontinence	
Fecal	Urinary
10	20

Type of injury					
3rd and 4th degree perineal tears	Vesico vaginal fistula	Circumferential fistula	Urethra-vaginal fistula	Rectovaginal fistula	Vesico-cervical fistula
5	13	6	1	4	1

Table 2 above shows that the majority (20 women) of the participants suffered urinary incontinence. Of the 30 study participants, five had incontinence of stool due to 3rd and 4th degree perineal tears so 25 were actual fistula cases.

Only eight participants had tried to get treatment of their fistula before.

Surgical outcomes among the 25 clients repaired at Mashoko hospital

The outcome of surgery upon discharge was classified into three categories

1. Dry - fistula was successfully closed and there is no leaking
2. Incontinence - surgery was successful in closing the fistula hole but patient is discharged leaking due to stress incontinence
3. Fistula not closed – fistula was not closed (a dye test is done to distinguish leaking due to incontinence or unsuccessful surgery).

Those with perineal tears were repaired accordingly.

Fig 4 below shows the surgical treatment outcomes for fistula cases.

Fig 4: Treatment outcomes for fistula cases (n=25)

Fig 4 shows that 22 women, which were the majority of the participants went home dry. The fistula were closed and they no longer experienced incontinence, two participants had failed surgery, their fistula were not closed. The treatment success rate was 92%.

All participants were discharged from hospital without symptoms of Covid-19. No further diagnostic tests were done for Covid-19.

Discussion

The main fistula repair center in Zimbabwe, Chinhoyi Provincial Hospital stopped conducting fistula repair surgeries because of the Covid-19 pandemic. This was necessitated by the need to control the pandemic. Infection prevention and control measures which included social distancing, frequent hand sanitization and wearing of face masks were strictly observed. These measures meant cutting on most non-emergency surgeries so as to avoid unnecessary human contact and creating space for anticipated rise in Covid-19 case load. This was a trend noted in other hospitals. A study conducted in October 2020 at a tertiary UK hospital found that the hospital reduced non-emergency operations by 44% due to Covid-19 [5]. A study on the effect of Covid-19 pandemic on health services in Nigeria also found out that the pandemic had thrown health systems into disarray and most services which were deemed not life-saving were put on hold and most efforts directed to the fight of the pandemic [6].

The attendance rate of women to the repair camp was greatly affected by the Covid-19 pandemic. Most of the women (43%) came from Manicaland Province alone. This may be attributed to the increased efforts in active surveillance in the province. The introduction and scaling up of the community fistula surveillance program has increased the awareness of fistula as a treatable condition in Manicaland. Knowledge of obstetric fistula as a medical condition which can be treated and awareness of availability of treatment services has been shown to increase uptake of fistula repair services [7,8]. In a report of a two-week fistula campaign in Nigeria, the highly publicized event saw more than 500 fistula survivors being treated [9].

The fact that most fistula cases came from Manicaland may not necessarily mean that the province has the highest fistula burden in the country but awareness raising, active surveillance and publicizing of fistula repair program in the province may help explain this observation.

Active search for fistula cases was being done by community based workers, fistula survivors and local health workers and this may have helped the community to receive the information and respond better. This phenomenon was also noted in a study documenting the lived experiences of women with fistula in Kenya [10]. The researchers found that women opened up better to locally based health workers and local community workers.

As noted in other studies, a significant proportion of participants were adolescent and young women aged 24 years and below [11]. In a study conducted in Kaptembwa-Nakuru in Kenya it was noted that only 2.04% of the responds were above 25 years of age [11].

In their study documenting the experiences of women living with obstetric fistula in 2008, Semere and Nour argued that the body of a girl below the age 20 years is not mature enough to withstand the burden of child bearing, therefore they are at increased risk of developing fistula [12]. Contrary to this finding, a study on fistula survivors' experiences in Ghana had only one for the ten participants below 25 years [8]. These differences could be due to sampling issues. However, the World Health Organization (2019) and the United Nations Population Fund (2015) recognize childbirth during tender ages below age 25 years as high risk for developing obstetric fistula [1,4].

The findings of this study revealed that most participants had developed fistula 5 years or less prior to data collection for this study, with the majority of the participants having developed the problem less than 1 year ago. This finding is of concern as it shows that obstetric fistula continues to be a significant complication of pregnancy in Zimbabwe. The country has one district hospital (comprehensive emergency obstetric and neonatal care facility) for every 250,000 population (13). This is twice the recommended level for such facilities of 1 per 500,000 population (14). In theory, Zimbabwe should have very few or no obstetric fistula cases. This observation has a reference to the strength and effectiveness of the national health system as noted by other studies and the WHO (2014) that the continued occurrence of obstetric fistula is an indication of weak health systems [13–15].

Although there were other means of participants' referral to the fistula center, the majority (20 out of 28) were referred by community based workers in Manicaland through the community surveillance system. The majority of the women identified and referred through this system actually had fistula i.e. 19 of the 20. Only one had stool incontinence due to a 4th degree perineal tear. Although it may be too soon and the numbers inadequate to evaluate the specificity of the community fistula screening tool, these results show promising specificity thresh hold of the tool.

This study noted that most of the participants had vesico-vaginal fistula and most suffered urinary incontinence, this is in agreement with findings of other studies and documented

literature [16]. The high treatment success rate observed by this study, 92%, gives hope to fistula survivors for restoration of good health and dignity after repair.

Other studies also noted that when surgical repair of fistula is performed by experienced surgeons, the treatment success rates are very high (18,19)] This observation is believed to motivate fistula survivors to take up e treatment services especially if more survivors who had successful repair advocate for treatment.

Conclusions

With the above findings, this study concluded that the Covid-19 pandemic negatively affected the fistula repair program in the country as it was considered one of the non-essential services. Women living with obstetric fistula are there in communities as shown by the Manicaland case, they need to be identified, given information on available treatment services and linked to fistula care programs for repair, even during the Covid-19 pandemic.

It also concluded that community based workers and fistula survivors can be instrumental in community surveillance of fistula cases in their communities when they are trained on how to use standard surveillance tools.

Recommendations

Basing on the findings of this study, it is recommended to the Ministry of Health and Child Care to scale up and intensify fistula case finding in communities and link them to treatment services so as to end or minimize suffering of women and girls. There is need to increase treatment centers so that patients will not need to travel long distances to access services, this need has been highlighted by the Covid-19 lockdown. It is also recommended to scale up the community obstetric fistula surveillance system making use of community based workers and other personnel to actively search for fistula cases in the community. Use of mobile communications through phone calls or social network platforms to sustain screening and identification of women living with fistula so as to have them repaired as soon as possible after the Covid-19 pandemic or when the situation allows.

Acknowledgements

The authors acknowledge contributions of Sandra Murwira for assisting in conducting data collection from the key informants.

References

1. UNFPA. The Maternal Health Thematic Fund 2014 Annual Report: Improving maternal health, surging toward the 2015 deadline [Internet]. 2015 [cited 2019 Jun 1]. Available from: https://www.unfpa.org/sites/default/files/pub-pdf/MHTF%20annual%20report%20for%20WEB_0.pdf

2. Kay A, Nishimwe A, Star Hampton B. Giving Voice to the Experiences of Rwandan Women With Urogenital Fistula. Ann Glob Health. 2016 Mar 29;81(5):636.

3. Mselle LT, Kohi TW. Living with constant leaking of urine and odour: thematic analysis of socio-cultural experiences of women affected by obstetric fistula in rural Tanzania. BMC Womens Health. 2015 Dec;15(1):107.

4. The World Health Organization W. 10 Facts on Obstetric Fistula [Internet]. Geneva; 2019 Jan [cited 2019 May 30]. Available from: https://www.who.int/features/factfiles/obstetric_fistula/en/

5. Wade S, Nair G, Ayeni HA, Pawa A. A Cohort Study of Emergency Surgery Caseload and Regional Anesthesia Provision at a Tertiary UK Hospital During the Initial COVID-19 Pandemic. Cureus. 2020 Jun;12(6):e8781.

6. Ajibo H. Effect of Covid-19 on Nigerian Socio-economic Well-being, Health Sector Pandemic Preparedness and the Role of Nigerian Social Workers in the War Against Covid-19. Soc Work Public Health. 2020 Sep 1;35(7):511–22.

7. Khisa AM, Omoni GM, Nyamongo IK, Spitzer RF. 'I stayed with my illness': a grounded theory study of health seeking behaviour and treatment pathways of patients with obstetric fistula in Kenya. BMC Womens Health. 2017 Dec;17(1):92.

8. Abokaiagana A. Experiences of women with Obstetric fistula in the Bawku East District of the Upper East Region. :155.

9. Ramsey K, Iliyasu Z, Idoko L. Fistula Fortnight: Innovative partnership brings mass treatment and public awareness towards ending obstetric fistula. Int J Gynecol Obstet. 2007;99(S1):S130–6.

10. Khisa AM, Nyamongo IK. Still living with fistula: an exploratory study of the experience of women with obstetric fistula following corrective surgery in West Pokot, Kenya. Reprod Health Matters. 2012 Mar;20(40):59–66.

11. Kimani ZM, Ogutu O, Antony Kibe. The Prevalence and Impact of Obstetric Fistula on Women of Kaptembwa – Nakuru, Kenya. Int J Appl Sci Technol. 2014;4(3):15.

12. Semere L, Nour NM. Obstetric Fistula: Living With Incontinence and Shame. Rev Obstet Gynecol. 2008;1(4):193–7.

13. Ministry of Health and Child Care, Zimbabwe. National Health Strategy for Zimbabwe 2016-2020. 2016.

14. The World Health Organization. Obstetric Fistula guiding principels for clinical managent and programme development [Internet]. WHO Press, Geneva; 2017 [cited 2020 May 1]. Available from: https://www.afro.who.int/sites/default/files/2017-06/mps%20Fistula2.pdf

15. Ijaiya MA, Aboyeji PA. Obstetric urogenital fistula: the Ilorin experience, Nigeria. West Afr J Med. 2004;23(1):7–9.

16. Kimani ZM, Ogutu O, Kibe A. The prevalence and impact of obstetric fistula on women of Kaptembwa–Nakuru, Kenya. Int J Appl. 2014;4(3):273–87.

17. Okoye UO, Emma-Echiegu N, Tanyi PL. Living with vesico-vaginal fistula: experiences of women awaiting repairs in Ebonyi State, Nigeria. Tanzan J Health Res. 2014;16(4):322–8.

18. Hebbar S, Pandey H, Chawla A. Understanding King's Health Questionnaire (KHQ) in assessment of female urinary incontinence. Int J Res Med Sci. 2015;3(3):531–8.

19. Wall LL, Karshima JA, Kirschner C, Arrowsmith SD. The obstetric vesicovaginal fistula: characteristics of 899 patients from Jos, Nigeria. Am J Obstet Gynecol. 2004;190(4):1011–6.

Fig 1

Click here to access/download;Figure;Figure 1.tif

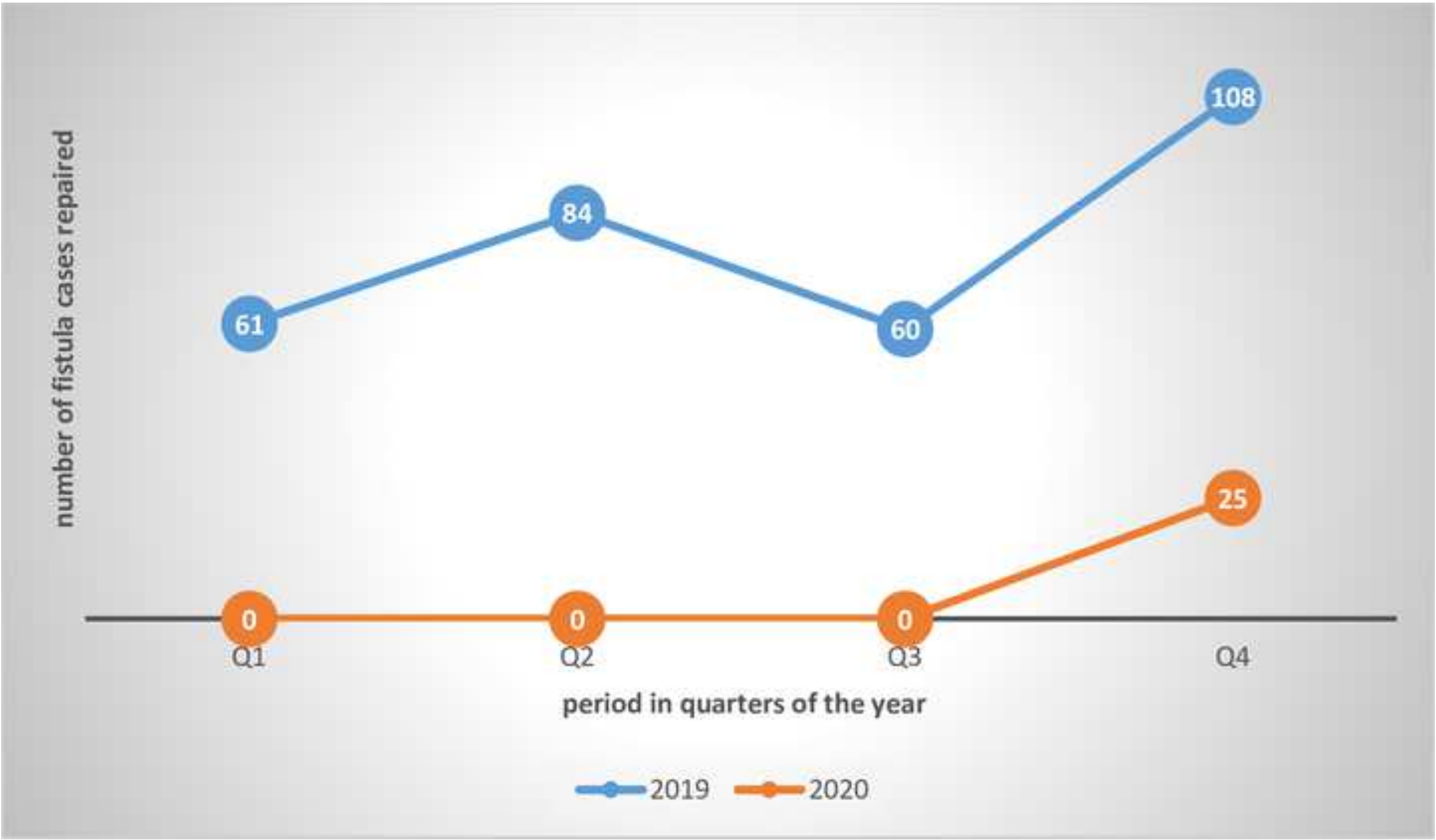

Fig 2

Click here to access/download;Figure;Figure 2.tif

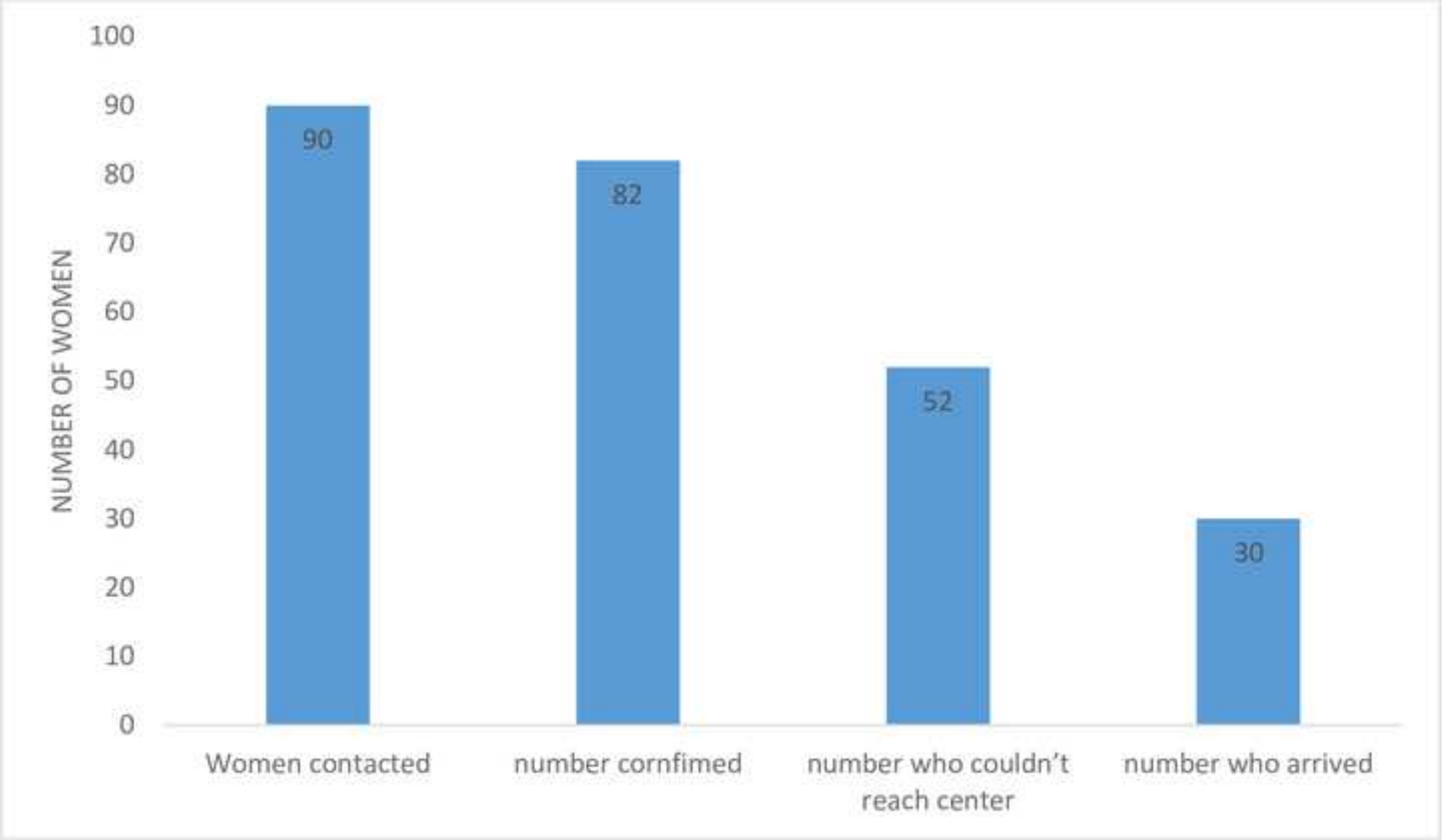

Fig 3

Click here to access/download;Figure;Figure 3.tif

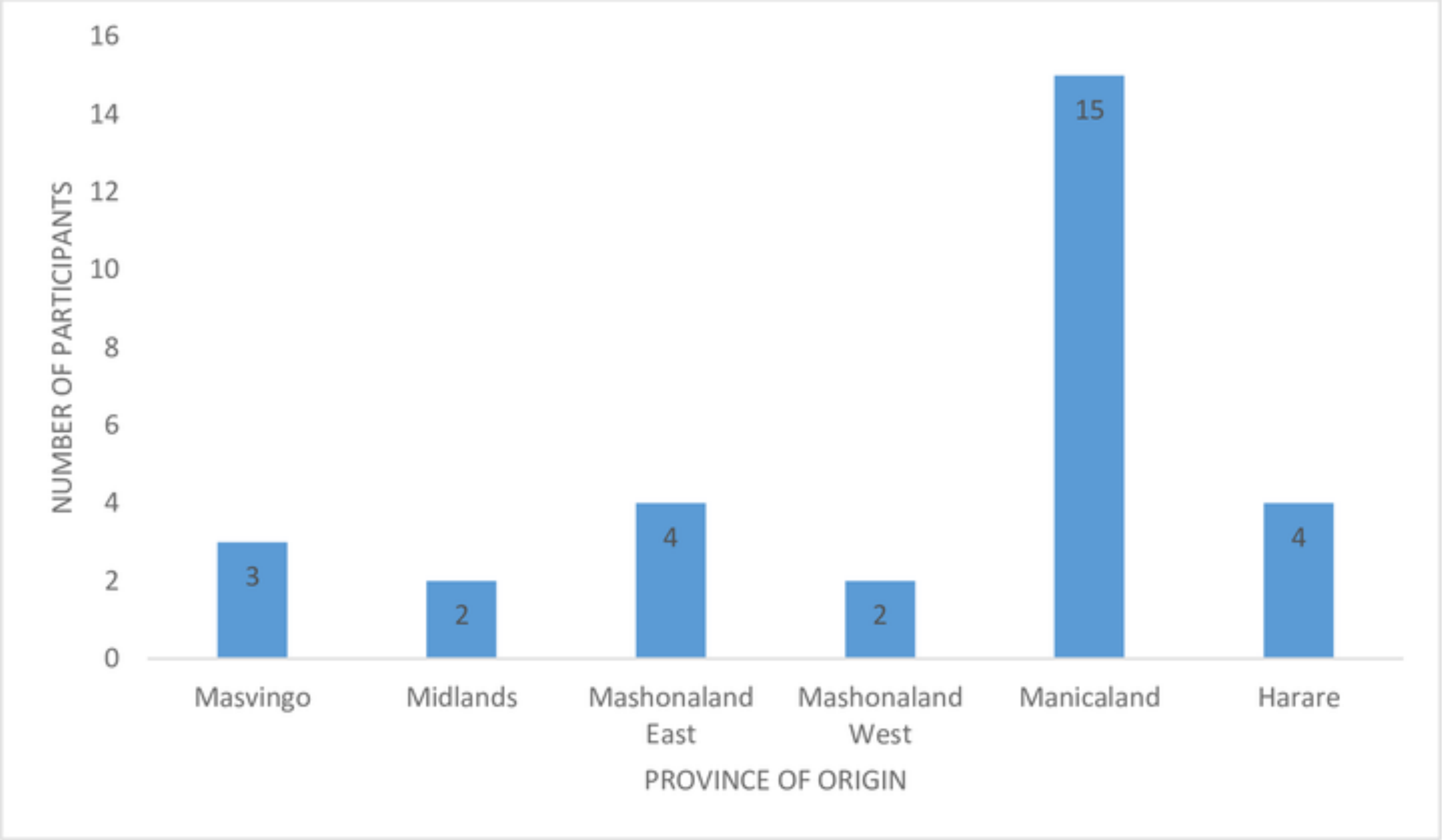

Fig 4

Click here to access/download;Figure;Figure 4.tif

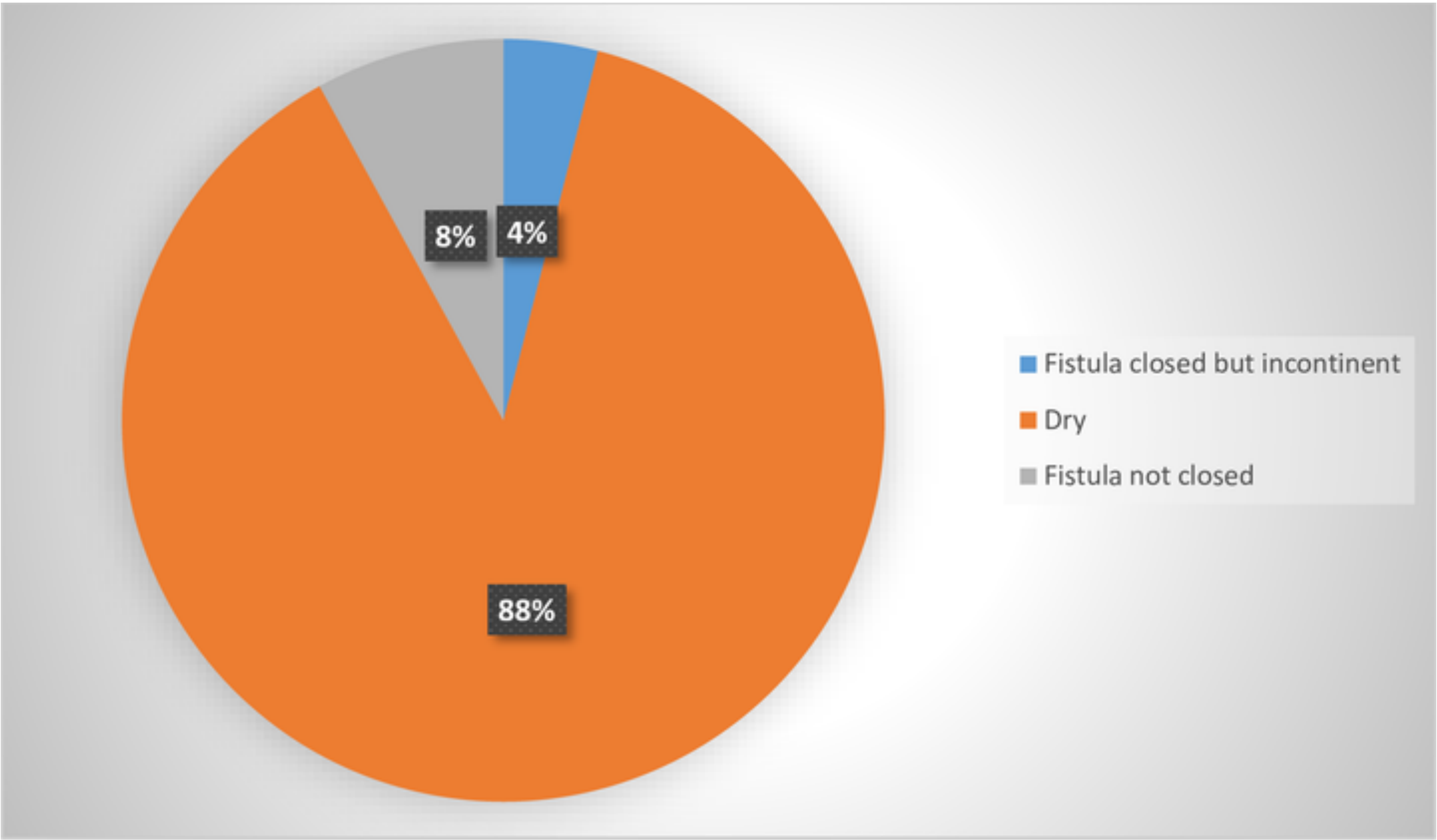

S1 Key informant indepth interview guide

Click here to access/download
Supporting Information
Key informant indepth interview guide.docx

S2 Q4 2020 Fistula repair logsheet, Mashoko Christian Hospital

Click here to access/download
Supporting Information
Mashoko Q4 2020 fistula log.xlsx
