# Supplementary material for: Impact of Covid-19 pandemic on obstetric fistula repair program in Zimbabwe
Source: PLoS One. 2021 Apr 1;16(4):e0249398. doi: 10.1371/journal.pone.0249398 (PMC8016295; doi:10.1371/journal.pone.0249398)
Supplement: Attachment — Submitted filename: Response to reviewers.docx [file pone.0249398.s004.docx]

Chipo Chimamise
Africa University
P.O. Box 1320, Mutare,

Zimbabwe

15 March 2021

Dear Editor

Plos One Journal

**RE: Revision of manuscript for publication in Plos One Journal: PONE-D-21-05832**

Thank you for your valuable comments. The authors have edited and corrected accordingly. Please see point by point detailed responses below.

- Please review your reference list to ensure that it is complete and correct. If you have cited papers that have been retracted, please include the rationale for doing so in the manuscript text, or remove these references and replace them with relevant current references. Any changes to the reference list should be mentioned in the rebuttal letter that accompanies your revised manuscript. If you need to cite a retracted article, indicate the article’s retracted status in the References list and also include a citation and full reference for the retraction notice.

Reference list reviewed, the reference which was not complete was corrected ie ref 13. Abokaiagana A. Experiences of women with Obstetric fistula in the Bawku East District of the Upper East Region, Ghana [Internet]. University of Ghana; 2010 [cited 2021 Jan 18]. Available from: <http://ugspace.ug.edu.gh>.

It was originally cited as: 8.	Abokaiagana A. Experiences of women with Obstetric fistula in the Bawku East District of the Upper East Region. :155.

- Please ensure that your manuscript meets PLOS ONE's style requirements, including those for file naming.

Figure file naming was corrected. Fig 1was renamed Fig 1.tif, Fig 2 was renamed Fig 2.tif, Fig 3 was renamed Fig 3.tf and Figure 4 was renamed Fig 4.tif

- Please address all comments from reviewers

This was done

- Please beef up the section on weaknesses of the study

# This was attended to:

# **Study limitations**

Data collection was mainly records review and telephone conversations, this approach poses restrictions on collecting the finer details on information obtained as compared to face-to-face interviews. Also, a larger sample size would have provided more confidence in obtained results.

- Please include a section on recommendations to programmers and policymakers

This was addressed:

# **Recommendations to programmers and policy makers**

Basing on the findings of this study, it is recommended to the Ministry of Health and Child Care to scale up and intensify fistula case finding in communities and link them to treatment services so as to end or minimize suffering of women and girls. There is need to increase treatment centers so that patients will not need to travel long distances to access services, this need has been highlighted by the Covid-19 lockdown. It is also recommended to scale up the community obstetric fistula surveillance system making use of community based workers and other personnel to actively search for fistula cases in the community. The use of mobile communications through phone calls or social network platforms is recommended to sustain screening and identification of women living with fistula so as to have them repaired as soon as possible after the Covid-19 pandemic or when the situation allows. The program needs to be expanded to establish fistula repair centers in other provinces for equity and accessibility even in times like the Covid-19 pandemic lockdown.

- Reviewer #1: This is a very good manuscript that attempts to describe the impact of the COVID-19 pandemic on obstetric fistula repair in Zimbabwe. Obstetric fistulae remain a significant challenge in Zimbabwe and attempts to repair them are commendable. This is a technically sound, original manuscript, which has a significant contribution to the literature regarding the impacts of the COVID-19 pandemic on public health services delivery in a resource-limited setting. This study is worthy publishing; however, the author must correct the many grammatical errors noted in the manuscript, which are highlighted on the attachment.

This was attended to as highlighted in the copy with track changes

- Reviewer #2: The manuscript is a relevant piece of academic writing, however the statistical analysis is too simplistic as the authors chose to limit themselves to univariate analysis. Beyond the reasons provided by the clients one might want to know whether there were any factors associated with a no show for fistulae repair(only 1/3 came)? Is it possible that clients with certain characteristics were more likely not to show up for repair than others? These questions would give insights into issues of equity which are relevant for such a program undertaking. Such questions would have been answered through multivariate analysis and a sample size of 90 must be powered enough to give insight. Given that a complete dataset is available I suggest that the authors consider adding a more detailed statistical analysis beyond the descriptives they shared

Attended to. Authors went further to perform bivariate analysis (only one factor was statistically significant) and discussed the findings in the discussion section and cascaded it to recommendations.

**In results:**

**Likelihood of attending the camp by province of origin**

At 95% confidence level, participants from Manicaland and Masvingo provinces were about 11 times more likely to reach the repair center than those coming from other provinces (OR 11.5; CI 3.7-35.3) and this difference was statistically significant (p<0.001), (Masvingo and Manicaland were put together because although Mashoko Hospital is in Masvingo, it is at the far border separating the two provinces).

**Likelihood of attending the camp by age group**

Adolescent and young participants (below age 25 years) were 0.3 times more likely to attend the camp than those 25years and older (OR1.3; CI 0.53-3.32) but this was not statistically significant (p=0.54).

Similar results were observed for period living with fistula and the likelihood of attending the camp where the OR was 1.06, CI 0.48-4.7, p = 0.8.

**In discussion:**

Proximity to the fistula repair center was the main determinant for whether the participants would reach the center or not. This may be so due to the number of law enforcement agents checkpoints participants had to pass on their way to hospital. Distance to health facilities which offer services was also noted by the World Health Organization to affect uptake of services [15]. This was made more evident by the Covid-19 pandemic lockdown restrictions.

**In recommendations:**

The program needs to be expanded to establish fistula repair centers in other provinces for equity and accessibility even in times like the Covid-19 pandemic lockdown.
